# Supplementary material for: Therapeutic Modifications without Discontinuation of Atezolizumab Plus Bevacizumab Therapy Are Associated with Favorable Overall Survival and Time to Progression in Patients with Unresectable Hepatocellular Carcinoma
Source: Cancers (Basel). 2023 Mar 2;15(5):1568. doi: 10.3390/cancers15051568 (PMC10001232; doi:10.3390/cancers15051568)
Supplement: Supplementary file 1 [file cancers-15-01568-s001.zip › cancers-2179105-supplementary.pdf]

Supplementary Materials

Therapeutic Modifications without Discontinuation of Atezolizumab plus Bevacizumab Therapy Are Associated with Favorable Overall Survival and Time to Progression in Patients with Unresectable Hepatocellular Carcinoma

Table S1. Cox regression analyses of factors contributing to OS in the patients who progressed on Atezolizumab and Bevacizumab therapy (*n* = 66)

Factors contributing to OS	Median OS (months)	Uni	Multivariate	
		p-value	Hazard ratio (95% CI)	P-value
Age				
<70 years	n.r	0.57		
≥70 years	14.70			
Sex				
Male	15.35	0.21		
Female	n.r.			
HBs antigen				
Positive	19.20	0.57		
Negative	15.35			
HCV antibody				
Positive	n.r.	0.41		
Negative	19.20			
Alcoholic liver disease				
Present	21.90	0.40		
Absent	15.35			
Esophageal varix				
Present	19.20	0.51		
Absent	n.r			
Gastric varix				
Present	19.20	0.68		
Absent	n.r			
History of curative therapy				
Present	n.r.	0.49		
Absent	15.35			
History of TACE				
Present	19.20	0.77		
Absent	10.98			

Child–Pugh class				
A	21.90	0.0020	reference	
B	7.30		3.83 (1.47–9.96)	**0.0060**
mALBI grade				
1	n.r.			
2a	19.20	0.41		
2b	15.35	0.21		
3	7.30	0.23		
ECOG-PS				
0	19.20	0.078		
1	9.63			
Maximum tumor size				
≤6 cm	21.90	0.010		0.27
>6 cm	8.58			
Tumor number				
≤6	n.r.	0.67		
>6	19.20			
Intrahepatic tumor volume				
<50%	19.20	0.0020	8.43 (1.90–37.46)	**0.0050**
≥50%	5.39		reference	
Macrovascular invasion				
Present	21.90	0.32		
Absent	11.47			
Extrahepatic spread				
Present	10.98	0.060		
Absent	21.90			
AFP concentration				
≥400 ng/mL	8.58	0.037		0.32
<400 ng/mL	21.90			
Number of chemotherapy lines				
First-line	21.90	0.82		
Second- or later-line	15.35			
Best response				
Objective response	n.r.	<0.001	0.11 (0.024–0.50)	**0.0040**
Stable disease	14.70	0.040	0.47 (0.17–1.32)	0.15
Progressive disease	5.39		reference	
Initial Bev dose				

Standard dose	11.24	0.39		
Reduced dose	19.20			
Experience of therapeutic modifications during TTP				
The discontinuation of both Atezo and Bev alone	5.66	0.50	2.37 (0.89–6.29)	0.083
The discontinuation of both Atezo and Bev with other therapeutic modifications†	7.92	0.56	0.46 (0.054–3.87)	0.47
Therapeutic modifications other than the discontinuation of both Atezo and Bev‡	n.r.	0.0010	0.24 (0.079–0.70)	**0.0090**
No therapeutic modification	7.46		reference	
IrAEs of any grade				
Experienced	21.90	0.66		
Not experienced	19.20			
AEs other than irAEs				
Grade ≥3	n.r.	0.028		0.82
Grade <2	11.47			
Subsequent therapy*				
Present	21.90	0.033		0.18
Absent	9.63			

Abbreviations: AEs, adverse events; AFP, alpha-fetoprotein; ALBI, albumin–bilirubin; Atezo, atezolizumab; Bev, bevacizumab; CI, confidence interval; DR, discontinuation rate; ECOG-PS, Eastern Cooperative Oncology Group performance status; HCV, hepatitis C virus; HBs, hepatis B surface; irAEs, immune-related adverse events; mOS, median overall survival; mTTP, median time to progression; n.r., not reached; OR, objective response; OS, overall survival; TACE, transarterial chemoembolization; TTP, time to progression; Uni, univariate. †Other therapeutic modifications include interruption of Bev, discontinuation of Bev and interruption of both Atezo and Bev. ‡Other therapeutic modifications than the discontinuation of both Atezo and Bev include a reduction in Bev, interruption of Bev, discontinuation of Bev, and interruption of both Atezo and Bev. *Thirty-one patients received subsequent therapy, including cabozantinib therapy (n = 1), the continuation of atezolizumab and bevacizumab therapy beyond progression (n = 16), hepatic arterial infusion chemotherapy (n = 4), lenvatinib therapy (n = 2), radiation therapy (n = 1), ramucirumab therapy (n = 2) and TACE (n = 5).
Bold font indicates significant p values.

Table S2. Cox regression analyses of factors contributing to (a) OS and (b) TTP in the patients with OR and SD (n = 86)

	(a) OS				(b) TTP			
Factors	Median OS (months)	Uni p-value	Multivariate Hazard ratio (95% CI)	p-value	Median TTP (months)	Uni p-value	Multivariate Hazard ratio (95% CI)	p-value
Age								
<70 years	21.90	0.27			5.16	0.091		

≥70 years	n.r.				9.01			
Sex								
Male	21.90	0.096			7.73	0.77		
Female	n.r.				8.06			
HBs antigen								
Positive	19.20	0.35			n.r.	0.046		0.28
Negative	21.90				7.33			
HCV antibody								
Positive	n.r.	0.44			5.75	0.23		
Negative	21.90				8.84			
Alcoholic liver disease								
Present	21.90	0.33			12.07	0.27		
Absent	n.r				7.33			
Esophageal varix								
Present	19.20	0.51			5.46	0.061		
Absent	n.r				10.00			
Gastric varix								
Present	19.20	0.68			12.07	0.78		
Absent	n.r				7.33			
History of curative therapy								
Present	n.r.	0.62			8.06	0.65		
Absent	21.90				6.81			
History of TACE								
Present	21.90	0.67			7.33	0.60		
Absent	n.r.				8.19			
Child–Pugh class								
A	n.r.	0.039			8.61	0.090		0.20
B	n.r.				4.96			
mALBI grade								
1	n.r.		reference		12.56		reference	
2a	21.90	0.62	2.26 (0.51-10.10)	0.29	7.33	0.22	n.a.	0.33
2b	n.r.	0.24	2.68 (0.67-10.68)	0.16	5.89	0.011	n.a.	0.69
3	7.30	0.019	129.14 (5.92-2815.88)	**0.002**	4.24	0.11	n.a.	**0.022**
ECOG-PS								
0	21.90	0.84			8.06	0.98		
1	n.r.				5.79			
Maximum tumor size								
≤6 cm	n.r	0.11			5.52	0.86		

>6 cm	n.r				8.06			
Tumor number								
≤6	n.r.	0.33			8.84	0.39		
>6	21.90				6.81			
Intrahepatic tumor volume								
<50%	21.90	0.011	36.88 (2.58–527.00)	**0.0080**	8.06	0.027	n.a.	**0.020**
≥50%	6.97		reference		2.66		reference	
Macrovascular invasion								
Present	19.20	0.056			4.41	0.13		
Absent	n.r.				8.61			
Extrahepatic spread								
Present	n.r.	0.20			9.01	0.61		
Absent	21.90				7.33			
AFP concentration								
≥400 ng/mL	19.20	0.20		0.19	5.16	0.26		
<400 ng/mL	n.r.				8.06			
Number of chemotherapy lines								
First-line	21.90	0.29			7.73	0.35		
Second- or later-line	19.20				8.06			
Best response								
Objective response	n.r.	<0.001	0.085 (0.018–0.40)	**0.0020**	10.00	<0.001	0.26 (0.14–0.47)	**<0.001**
Stable disease	15.35		reference		4.37		reference	
Initial Bev dose								
Standard dose	11.24	0.31			8.19	0.086		
Reduced dose	21.90				3.49			
Experience of therapeutic modifications during TTP								
The discontinuation of both Atezo and Bev alone	11.47	0.15	4.93 (1.37–17.74)	**0.015**	3.35	0.029	3.59 (1.57–8.23)	**0.0030**
The discontinuation of both Atezo and Bev with other therapeutic modifications[†]	n.r.	0.29	0.62 (0.070–5.51)	0.67	n.r.	0.065	0.37 (0.074–1.80)	0.22
Therapeutic modifications other than the discontinuation of both Atezo and Bev[‡]	n.r.	0.011	0.35 (0.081–1.45)	0.16	11.77	0.071	0.65 (0.30–1.42)	0.28
No therapeutic modification	15.35		reference		8.19		reference	
IrAEs of any grade								
Experienced	21.90	0.71			8.84	0.22		

Not experienced	n.r.				7.33	
AEs other than irAEs						
Grade ≥3	n.r.	0.16			6.87	0.81
Grade <2	21.90				8.84	

Abbreviations: AEs, adverse events; AFP, alpha-fetoprotein; ALBI, albumin–bilirubin; Atezo, atezolizumab; Bev, bevacizumab; CI, confidence interval; DR, discontinuation rate; ECOG-PS, Eastern Cooperative Oncology Group performance status; HCV, hepatitis C virus; HBs, hepatis B surface; irAEs, immune-related adverse events; mOS, median overall survival; mTTP, median time to progression; n.a., not applicable; n.r., not reached; OR, objective response; OS, overall survival; TACE, transarterial chemoembolization; TTP, time to progression; Uni, univariate. [†]Other therapeutic modifications include interruption of Bev, discontinuation of Bev and interruption of both Atezo and Bev. [‡]Other therapeutic modifications than the discontinuation of both Atezo and Bev include a reduction in Bev, interruption of Bev, discontinuation of Bev, and interruption of both Atezo and Bev. Bold font indicates significant *p* values.

Figure S1:Kaplan–Meier estimates of cumulative OS by (a) best response (n=100) and (b) response using landmark times at (b) 2 months (n=94), (c) 4 months (n=86) and (d) 6 months (n=73)

(a)

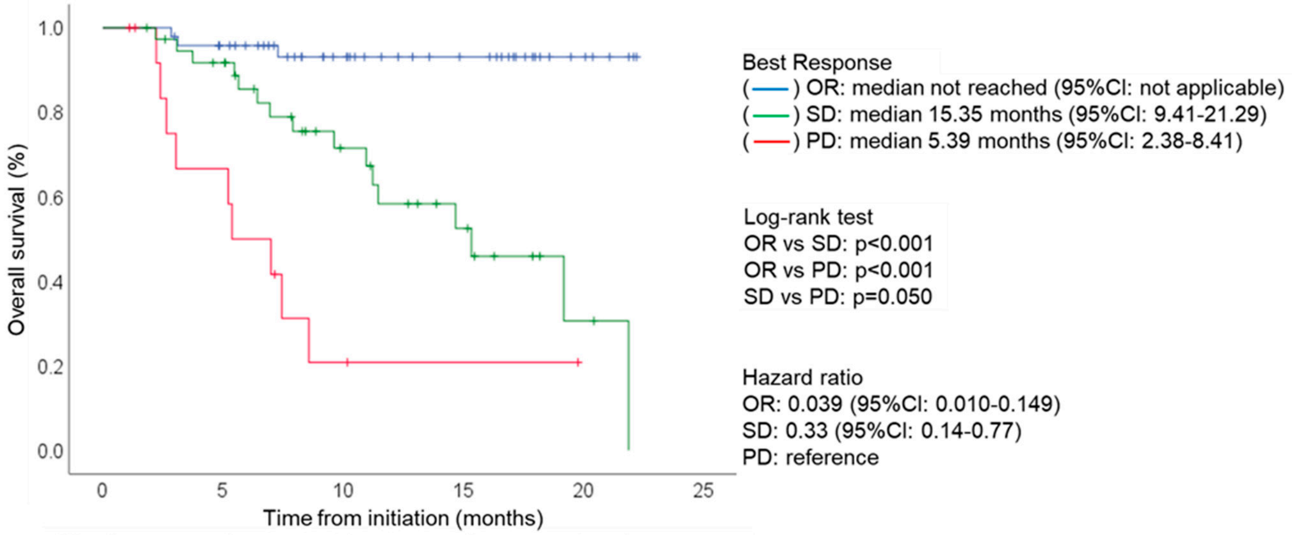

Number at risk							
	OR	48	43	28	18	3	0
	SD	38	32	17	9	2	0
	PD	14	8	2	1	0	

(b)

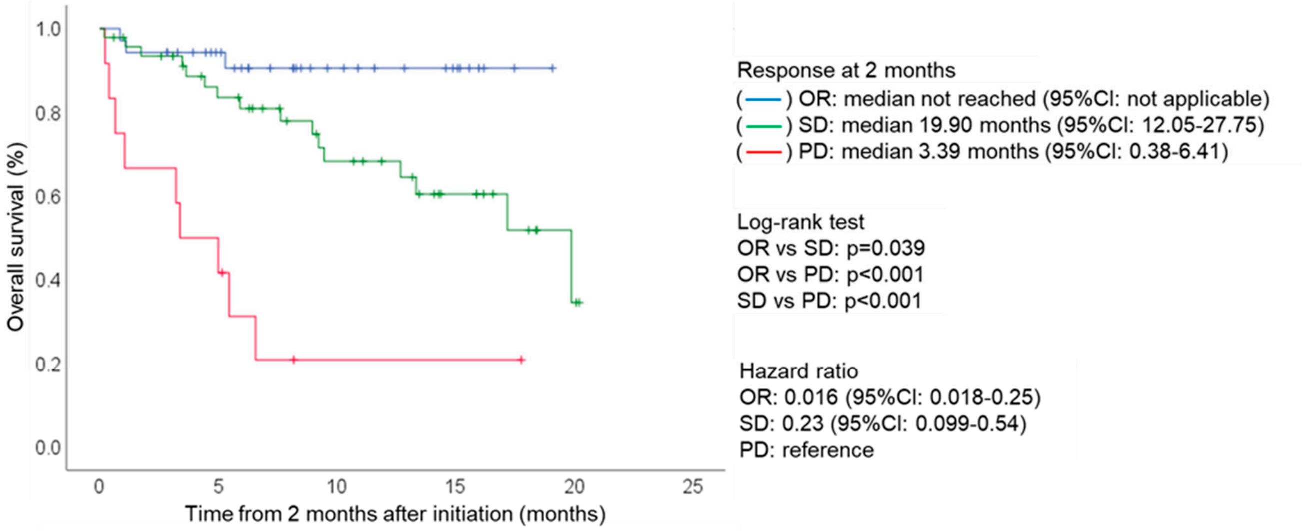

Number at risk							
	OR	35	26	13	7	0	
	SD	47	33	21	11	2 0	
	PD	12	6	1	1	0	

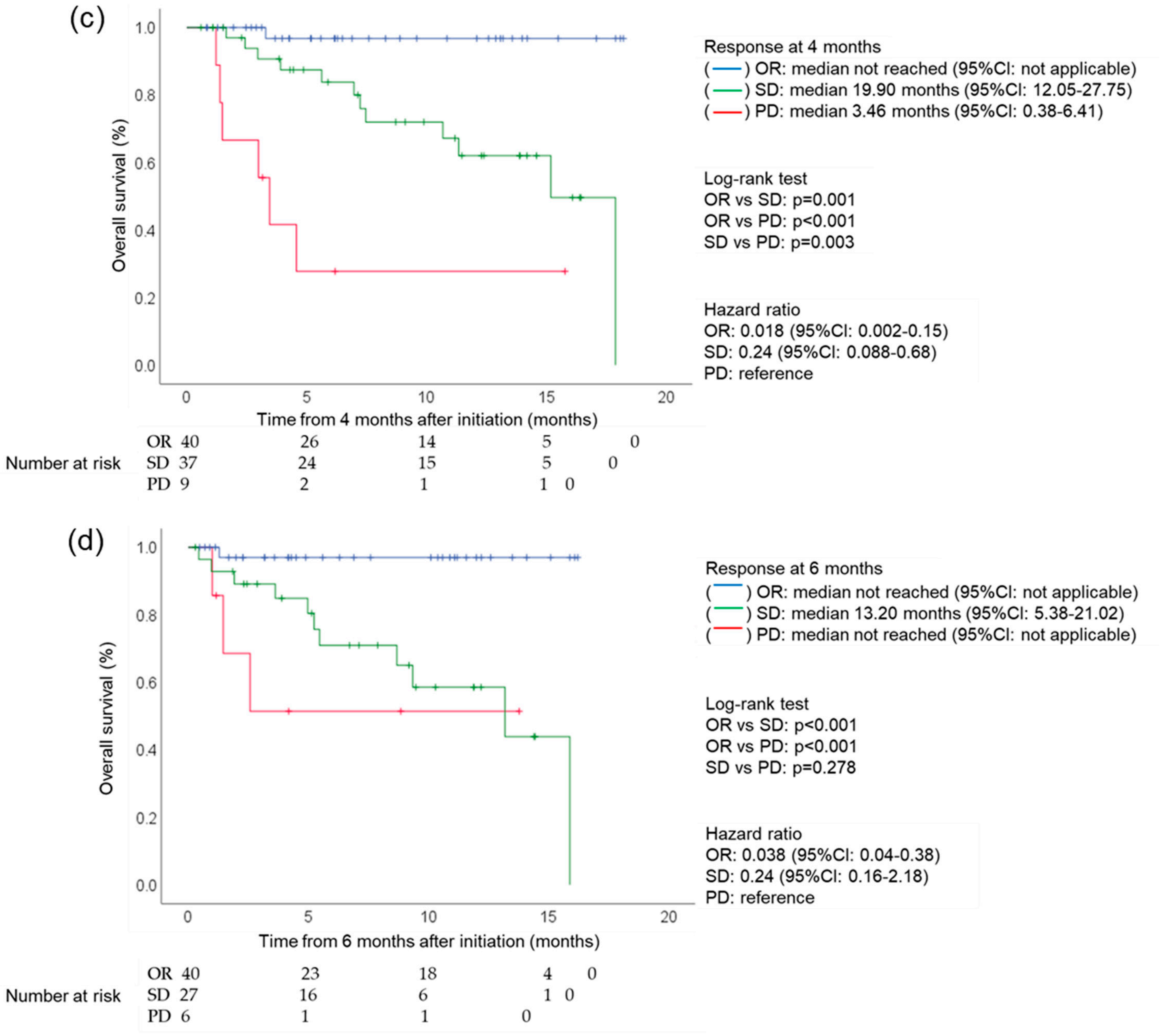
(c)
Overall survival (%)
1.0
0.8
0.6
0.4
0.2
0.0
0
5
10
15
20
Time from 4 months after initiation (months)
Response at 4 months
( ) OR: median not reached (95%CI: not applicable)
( ) SD: median 19.90 months (95%CI: 12.05-27.75)
( ) PD: median 3.46 months (95%CI: 0.38-6.41)
Log-rank test
OR vs SD: p=0.001
OR vs PD: p<0.001
SD vs PD: p=0.003
Hazard ratio
OR: 0.018 (95%CI: 0.002-0.15)
SD: 0.24 (95%CI: 0.088-0.68)
PD: reference
Number at risk
OR 40 26 14 5 0
SD 37 24 15 5 0
PD 9 2 1 1 0
(d)
Overall survival (%)
1.0
0.8
0.6
0.4
0.2
0.0
0
5
10
15
20
Time from 6 months after initiation (months)
Response at 6 months
( ) OR: median not reached (95%CI: not applicable)
( ) SD: median 13.20 months (95%CI: 5.38-21.02)
( ) PD: median not reached (95%CI: not applicable)
Log-rank test
OR vs SD: p<0.001
OR vs PD: p<0.001
SD vs PD: p=0.278
Hazard ratio
OR: 0.038 (95%CI: 0.04-0.38)
SD: 0.24 (95%CI: 0.16-2.18)
PD: reference
Number at risk
OR 40 23 18 4 0
SD 27 16 6 1 0
PD 6 1 1 0

Figure S2:Kaplan–Meier estimates of cumulative TTP by (a) best response (n=100) and (b) response using landmark times at (b) 2 months (n=81), (c) 4 months (n=60) and (d) 6 months (n=39)

(a)

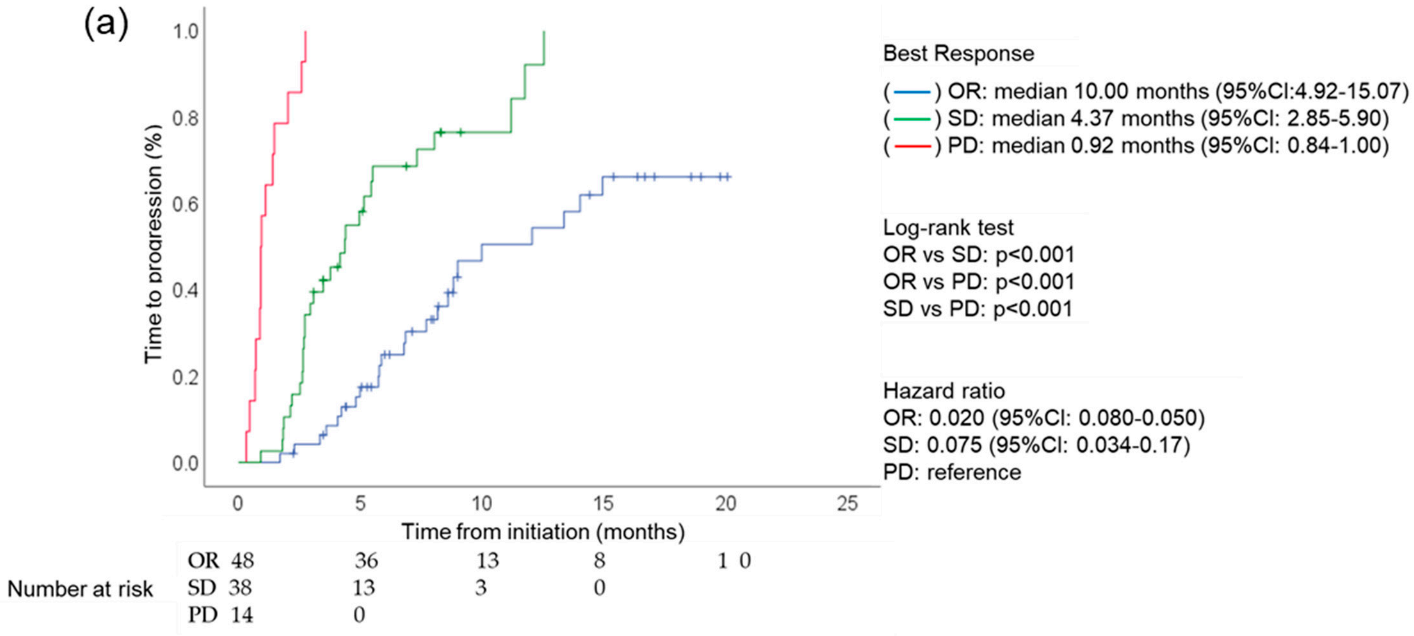

(b)

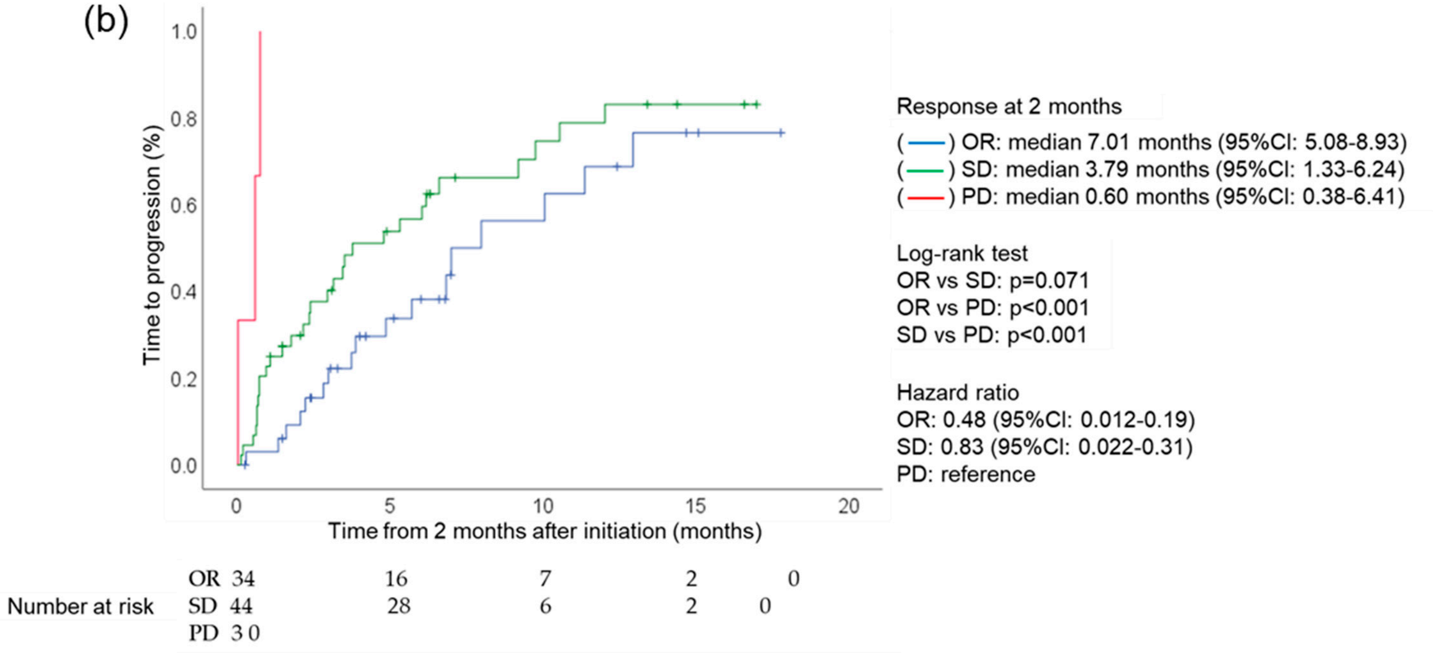

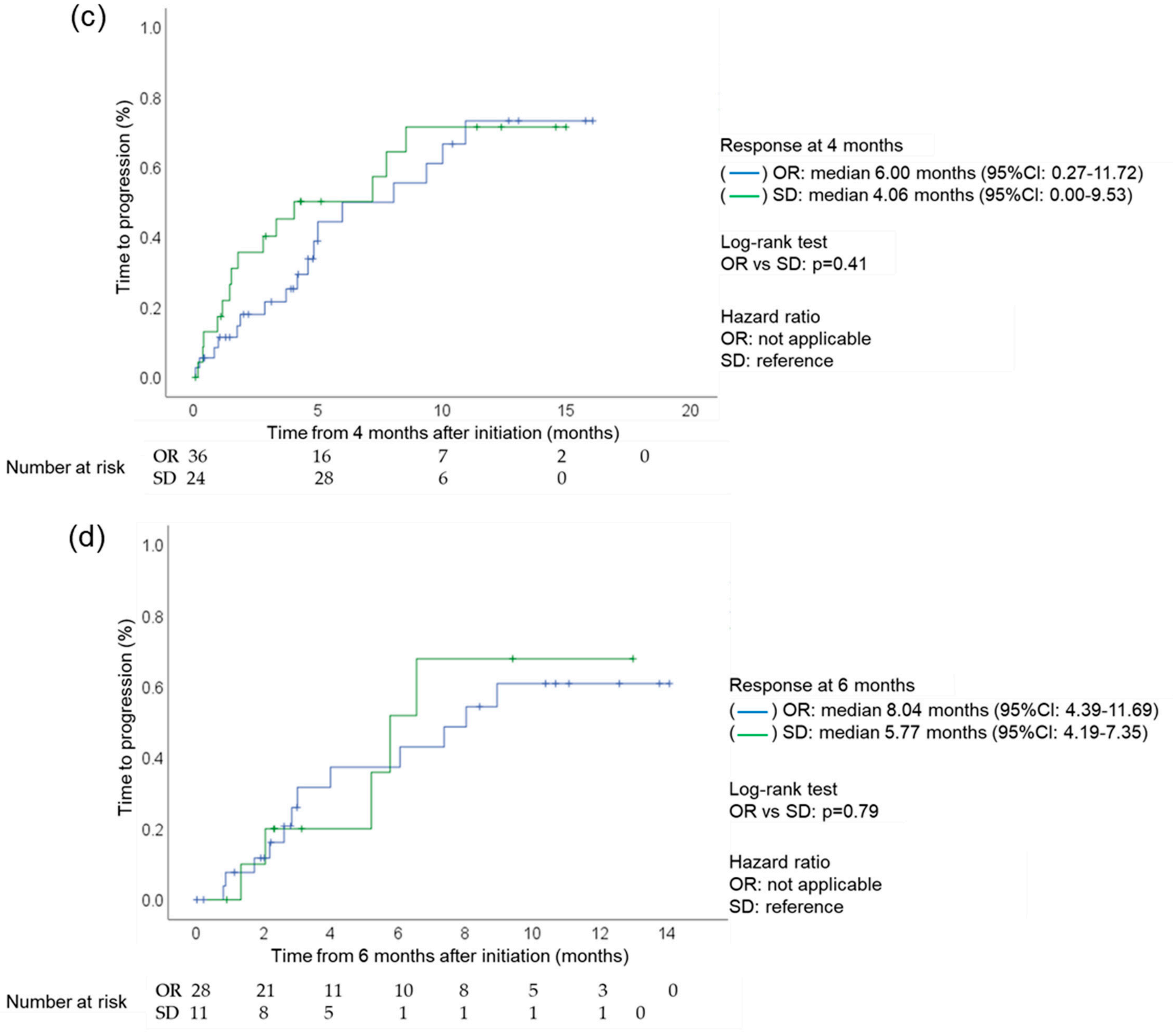
(c)
Time to progression (%)
1.0
0.8
0.6
0.4
0.2
0.0
0
5
10
15
20
Time from 4 months after initiation (months)
Response at 4 months
( ) OR: median 6.00 months (95%CI: 0.27-11.72)
( ) SD: median 4.06 months (95%CI: 0.00-9.53)
Log-rank test
OR vs SD: p=0.41
Hazard ratio
OR: not applicable
SD: reference
Number at risk
OR 36 16 7 2 0
SD 24 28 6 0
(d)
Time to progression (%)
1.0
0.8
0.6
0.4
0.2
0.0
0
2
4
6
8
10
12
14
Time from 6 months after initiation (months)
Response at 6 months
( ) OR: median 8.04 months (95%CI: 4.39-11.69)
( ) SD: median 5.77 months (95%CI: 4.19-7.35)
Log-rank test
OR vs SD: p=0.79
Hazard ratio
OR: not applicable
SD: reference
Number at risk
OR 28 21 11 10 8 5 3 0
SD 11 8 5 1 1 1 1 0

Figure S3:Kaplan–Meier estimates of cumulative OS by (a) therapeutic modifications during TTP (n=100) and (b) therapeutic modifications using landmark times at (b) 2 months (n=97), (c) 4 months (n=86) and (d) 6 months (n=73)

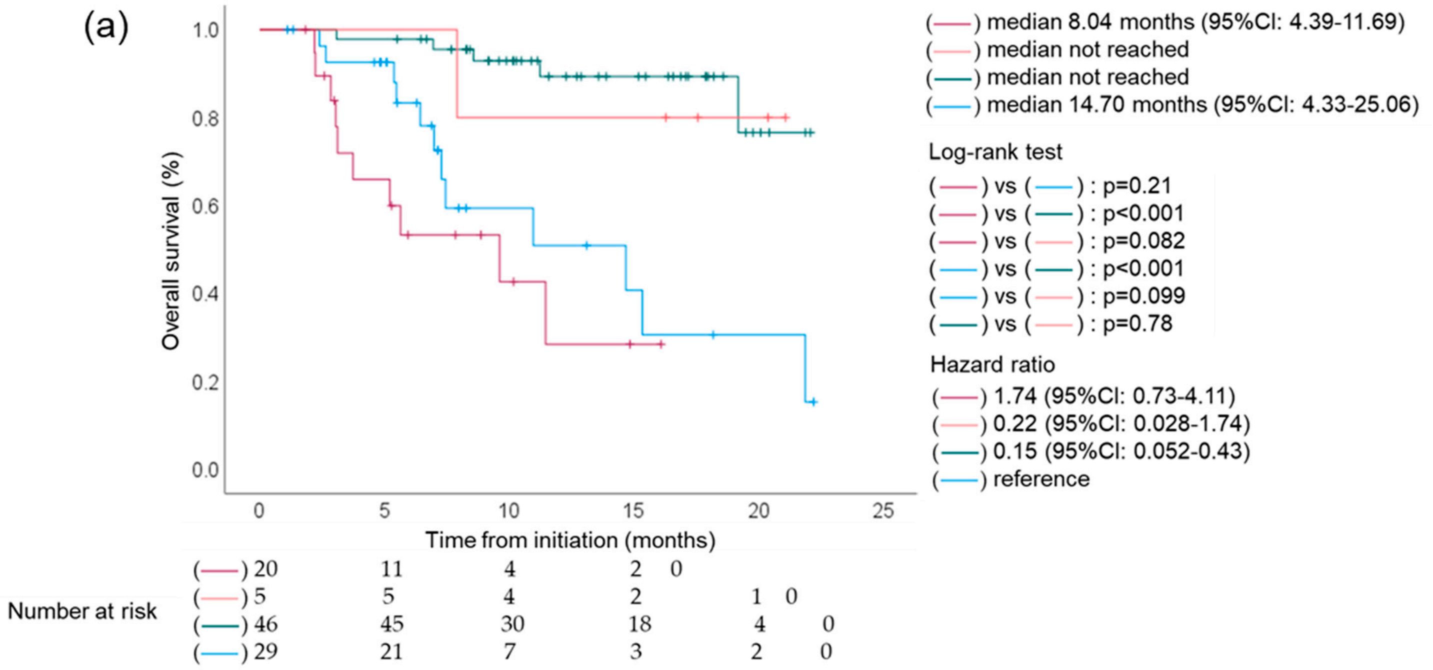

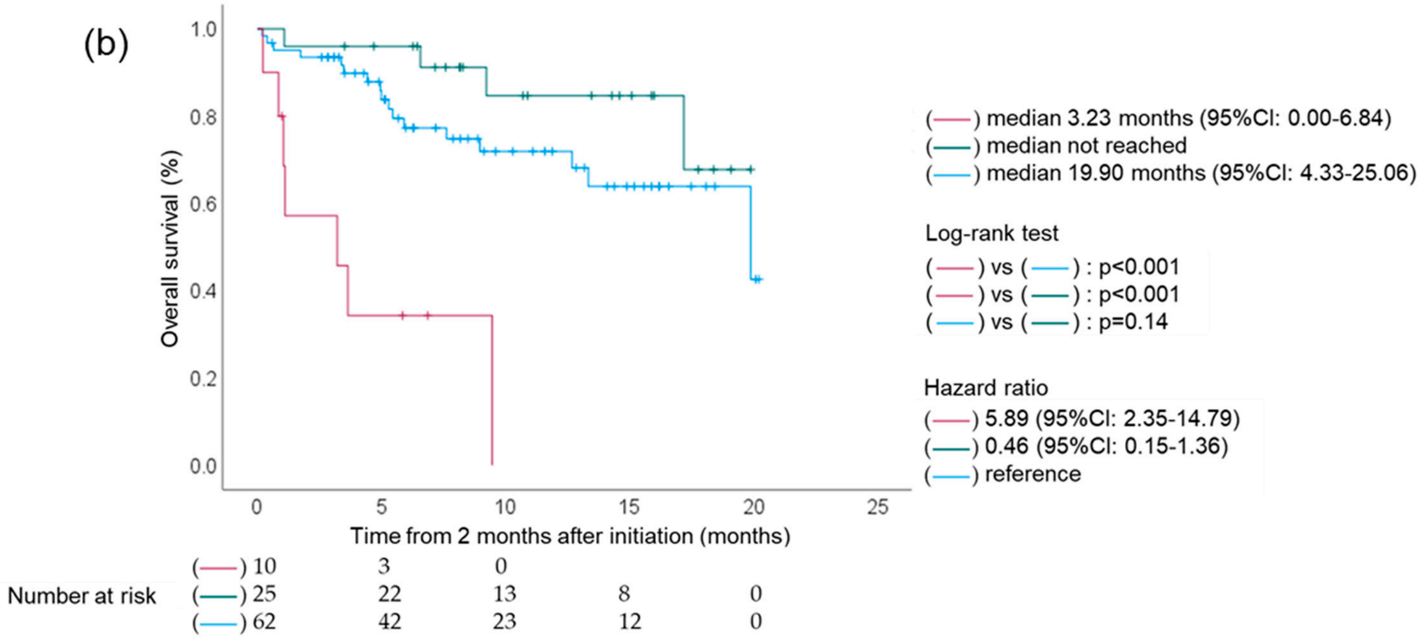

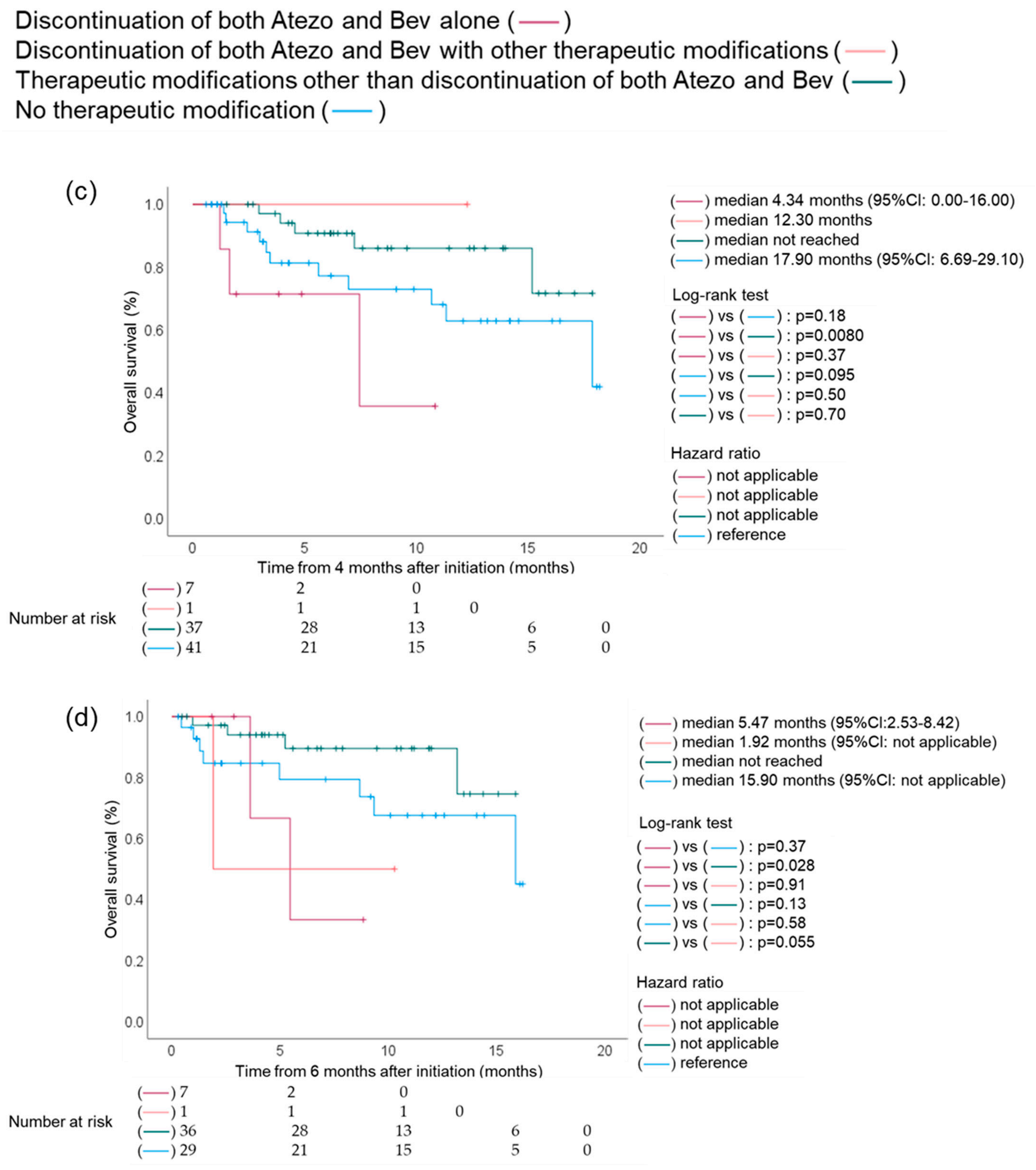
Discontinuation of both Atezo and Bev alone ( )
Discontinuation of both Atezo and Bev with other therapeutic modifications ( )
Therapeutic modifications other than discontinuation of both Atezo and Bev ( )
No therapeutic modification ( )
(c)
Overall survival (%)
1.0
0.8
0.6
0.4
0.2
0.0
0
5
10
15
20
Time from 4 months after initiation (months)
( ) median 4.34 months (95%CI: 0.00-16.00)
( ) median 12.30 months
( ) median not reached
( ) median 17.90 months (95%CI: 6.69-29.10)
Log-rank test
( ) vs ( ) : p=0.18
( ) vs ( ) : p=0.0080
( ) vs ( ) : p=0.37
( ) vs ( ) : p=0.095
( ) vs ( ) : p=0.50
( ) vs ( ) : p=0.70
Hazard ratio
( ) not applicable
( ) not applicable
( ) not applicable
( ) reference
Number at risk
( ) 7 2 0
( ) 1 1 1 0
( ) 37 28 13 6 0
( ) 41 21 15 5 0
(d)
Overall survival (%)
1.0
0.8
0.6
0.4
0.2
0.0
0
5
10
15
20
Time from 6 months after initiation (months)
( ) median 5.47 months (95%CI:2.53-8.42)
( ) median 1.92 months (95%CI: not applicable)
( ) median not reached
( ) median 15.90 months (95%CI: not applicable)
Log-rank test
( ) vs ( ) : p=0.37
( ) vs ( ) : p=0.028
( ) vs ( ) : p=0.91
( ) vs ( ) : p=0.13
( ) vs ( ) : p=0.58
( ) vs ( ) : p=0.055
Hazard ratio
( ) not applicable
( ) not applicable
( ) not applicable
( ) reference
Number at risk
( ) 7 2 0
( ) 1 1 1 0
( ) 36 28 13 6 0
( ) 29 21 15 5 0

Figure S4:Kaplan–Meier estimates of cumulative TTP by (a) therapeutic modifications during TTP (n=100) and (b) therapeutic modifications using landmark times at (b) 2 months (n= 84), (c) 4 months (n=60) and (d) 6 months (n=39)

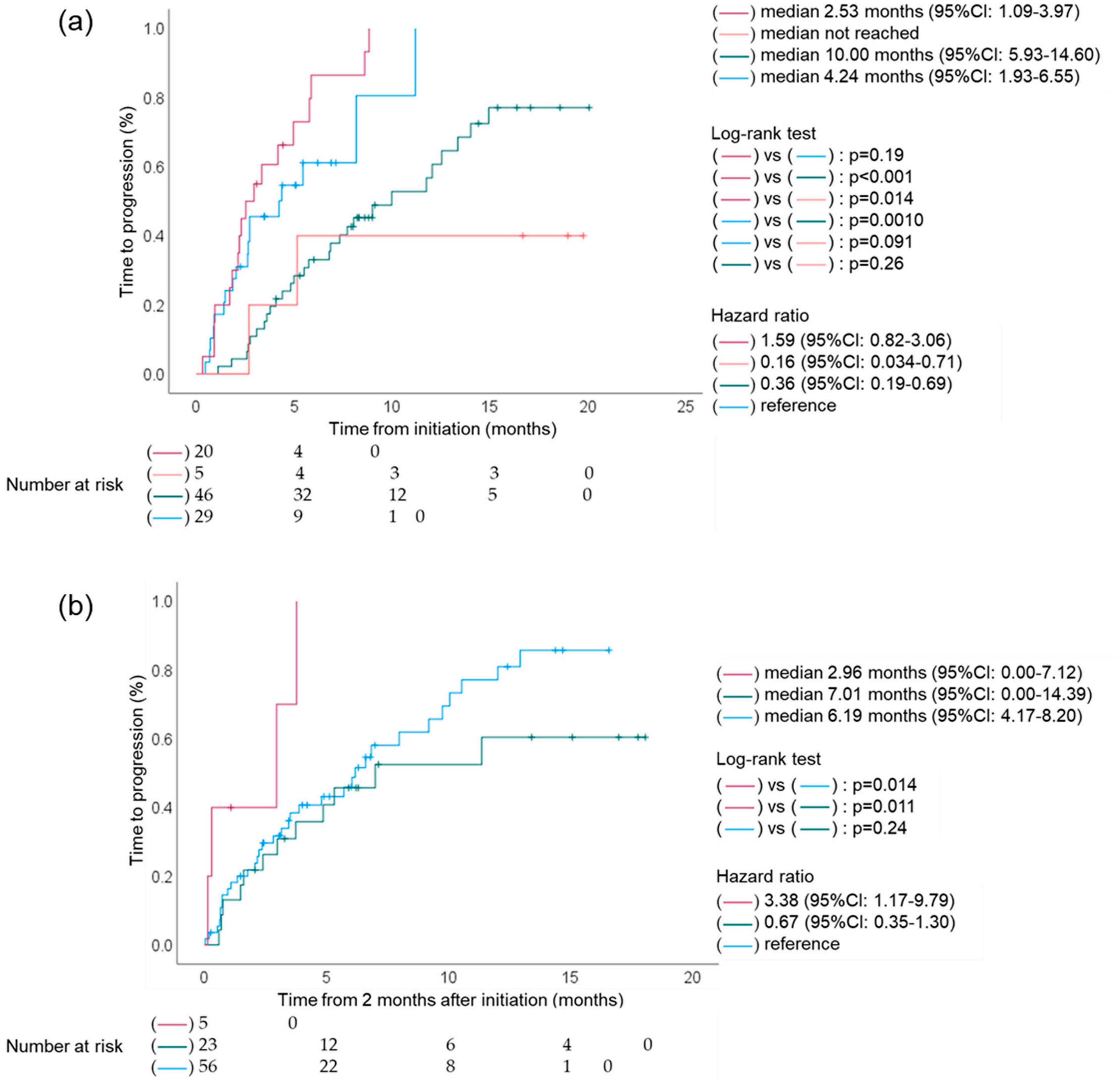

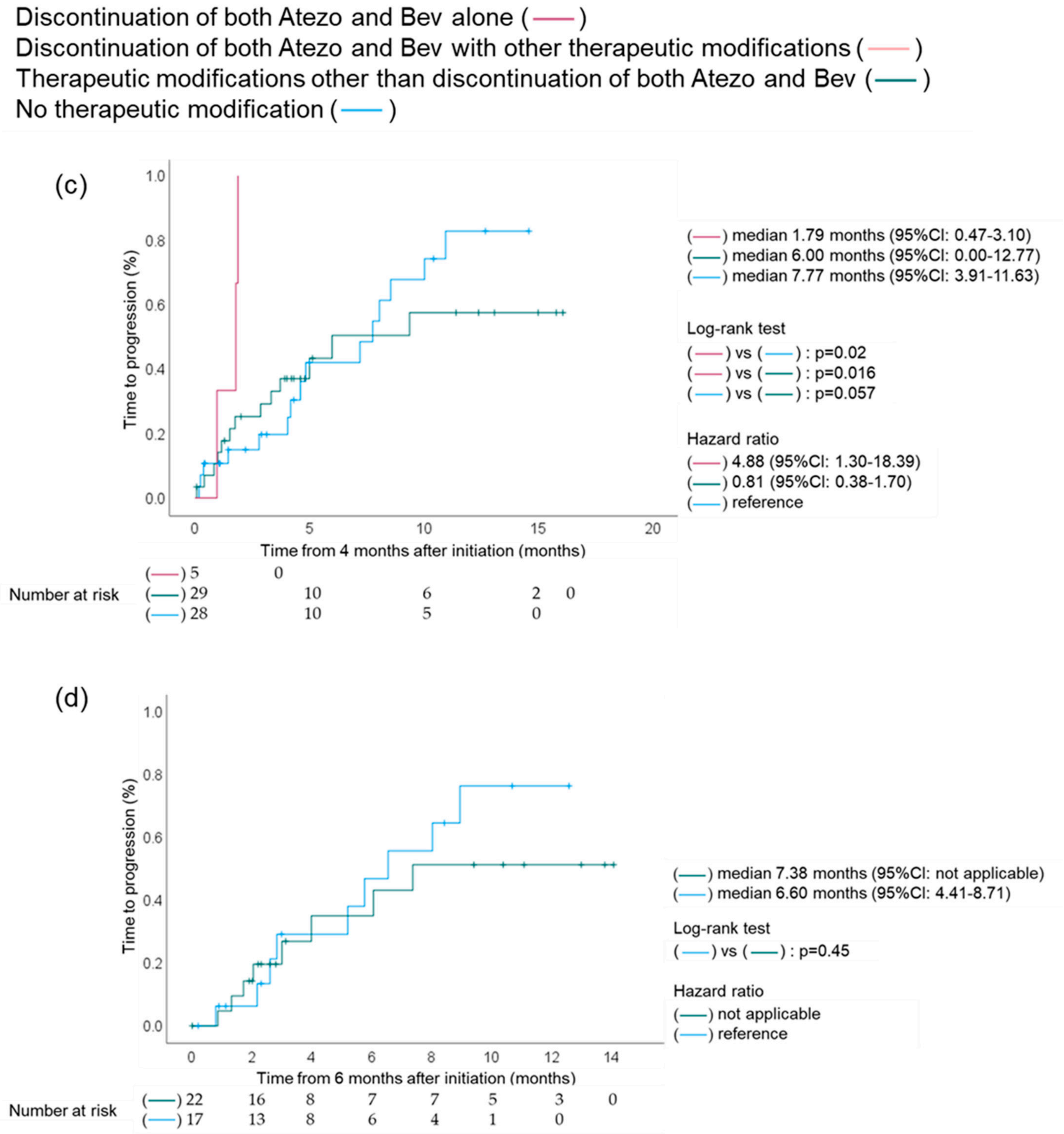
Discontinuation of both Atezo and Bev alone
Discontinuation of both Atezo and Bev with other therapeutic modifications
Therapeutic modifications other than discontinuation of both Atezo and Bev
No therapeutic modification
(c)
Time to progression (%)
Time from 4 months after initiation (months)
median 1.79 months (95%CI: 0.47-3.10)
median 6.00 months (95%CI: 0.00-12.77)
median 7.77 months (95%CI: 3.91-11.63)
Log-rank test
vs : p=0.02
vs : p=0.016
vs : p=0.057
Hazard ratio
4.88 (95%CI: 1.30-18.39)
0.81 (95%CI: 0.38-1.70)
reference
Number at risk
5 0
29 10 6 2 0
28 10 5 0
(d)
Time to progression (%)
Time from 6 months after initiation (months)
median 7.38 months (95%CI: not applicable)
median 6.60 months (95%CI: 4.41-8.71)
Log-rank test
vs : p=0.45
Hazard ratio
not applicable
reference
Number at risk
22 16 8 7 7 5 3 0
17 13 8 6 4 1 0
